# Dietary Patterns and Associated Factors Among Adolescents in Malaysia: Findings from Adolescent Nutrition Survey 2017

**DOI:** 10.3390/ijerph17103431

**Published:** 2020-05-14

**Authors:** Cheong Siew Man, Ruhaya Salleh, Mohamad Hasnan Ahmad, Azli Baharudin, Poh Bee Koon, Tahir Aris

**Affiliations:** 1 Institute for Public Health, National Institutes of Health, Ministry of Health Malaysia, Setia Alam 40170, Selangor, Malaysia; ruhaya.s@moh.gov.my (R.S.); hasnan.ahmad@moh.gov.my (M.H.A.); ps_azlibaharudin@moh.gov.my (A.B.); 2 Faculty of Health Sciences, Universiti Kebangsaan Malaysia, Kuala Lumpur 50300, Malaysia; pbkoon@ukm.edu.my; 3 Institute for Medical Research, National Institutes of Health, Ministry of Health Malaysia, Setia Alam 40170, Selangor, Malaysia; tahir.a@moh.gov.my

**Keywords:** dietary pattern, eating habits, food groups intake, adolescent nutrition

## Abstract

Balanced diet in the early stages of life plays a role in optimum growth and maintains good health status of adolescents. Dietary habits that are established during adolescence will sustain till adulthood. Therefore, this present study aims to identify the dietary patterns and to determine factors associated with dietary patterns in terms of socio-demographic characteristics, locality of schools, ethnicity, eating habits, self-perceived weight status, and food label reading habit among adolescents in Malaysia. Data from the Adolescent Nutrition Survey (ANS) 2017 was used for the present study. ANS is a population representative school-based cross-sectional study among school-going adolescents from primary four to secondary five from schools in 13 states and three federal territories registered under the Ministry of Education Malaysia. A self-administrated questionnaire was used to collect information on socio-demographic characteristics, locality of schools, ethnicity, eating habits, self-perceived weight status, and food label reading habit. A pre-tested face-to-face food frequency questionnaire (FFQ) was used to collect information on food group intake frequency. Dietary patterns were identified by using exploratory factor analysis and associated factors, using complex sample general linear model (GLM) analysis. All statistical analyses were carried out at 95% confidence interval or *p*-value < 0.05. The dietary patterns identified are healthy, unhealthy, and alternative proteins. The healthy dietary pattern was significantly associated with the types of school and ethnicity. The unhealthy dietary pattern was significantly associated with the locality of schools, ethnicity, frequency of snacks intake per week, frequency of eating out per week, self-perceived weight status, and food label reading habit. Significant associations were found between alternative proteins dietary pattern and locality of schools, ethnicity, and types of school. This study found that there is a disparity of dietary patterns between different ethnicity, locality of schools, and types of school. We recommend strategies of specifying ethnicity and geographical area to improve dietary patterns of adolescents in Malaysia.

## 1. Introduction

Previous nutritional epidemiology studies typically examined the relationship between nutrients and food groups’ intake and health outcomes [1,2]. However, people normally consume meals that consist of a combination of many different foods with different nutrient composition [3]. Therefore, the relationship between single nutrient and chronic diseases can be difficult to determine [4]. Dietary patterns analysis has become a recent interest because it describes the effect of diet on chronic diseases in a broader picture rather than focusing on specific nutrients [5].

Studies on the dietary patterns of adolescents have been conducted in several countries such as Australia [6], Brazil [7], Scotland [8], and China [9]. Various dietary patterns, namely eating habits around foods high in fat and sugar, vegetables, snacks, and traditional foods have been identified in these nationally representative studies. In general, unhealthy dietary patterns among adolescents were linked to higher risks of having metabolic syndrome, depression, and adulthood obesity [9,10,11,12]. Dietary patterns are also related to socio-demographic status. Inverse linear trends were observed between unhealthy dietary patterns and income level [13].

Furthermore, several smaller scale local studies have been conducted to investigate the determinants of dietary patterns among adolescents in certain states of Malaysia [14,15,16]. The determinants such as ethnicity, religion, household income, education level of parents has been identified in these local studies. Dietary patterns among adolescents is not associated with obesity [14]. However, high-energy dietary pattern has been shown to correlate with having lower cognitive ability among adolescents in a study carried out in a state located in Central Zone of Peninsular Malaysia [15]. Another study conducted in Kelantan state, a region at East Coast of Peninsular of Malaysia, revealed a significant difference in dietary patterns in different ethnicities [16].

There is still a lack of studies on population-based samples to provide a broader understanding for the association between dietary patterns and socio-demographic status in adolescents. Therefore, this study aims to identify the dietary patterns and their association to socio-demographic characteristics, locality of schools, ethnicity, eating habits, self-perceived weight status, and food label reading habit among adolescents in Malaysia. We wish that this study will help the policymakers and public health practitioners, the key elements of public health strategies, for non-communicable disease prevention in the young generation.

## 2. Materials and Methods

### 2.1. Study Design

Data for the present study came from the Adolescent Nutrition Survey (ANS) 2017—a school-based cross-sectional study among school-going adolescents aged 10 to 17 years. Data collection for the ANS was conducted from March to May 2017. A multi-stage stratified cluster sampling design was used to obtain a nationally representative sample of adolescents. The sampling frame consisted of all primary and secondary schools from 13 states and three federal territories registered under the Ministry of Education Malaysia. 

The first stage of sampling consisted of a random selection of schools (applying probability proportional to school enrolment size). A total of 311 schools (99 primary schools and 212 secondary schools) were selected to take part in this study. The second stage of sampling was a random selection of classes from each selected school. All students in the selected classes were eligible to participate in ANS and were given consent forms by their teachers prior to this study. Primary school students were given parents’ or caregivers’ consent forms and secondary school students were given self-administered consent forms. The third stage of sampling consisted of random selection of adolescents from each selected class to attend the face-to-face interviews of the food frequency questionnaire (FFQ).

### 2.2. Assessment Tools and Definition of Variables

A self-administered questionnaire with multiple choices questions in four languages (Malay, English, Chinese, and Tamil) was used to obtained information namely socio-demographic characteristics, dietary habits, and self-perceived weight status from the selected students. The survey was conducted anonymously and the information provided by the selected students remained confidential. Only data of students who provided complete information in FFQ were used for statistical analysis in this study.

The FFQ of ANS 2017 was adopted from the FFQ Malaysia School-based Nutrition Survey 2012 and the food items were modified according to the popularity of foods among adolescents. This FFQ contained 136 food items with eleven food groups namely cereals, grains, cereals products, and tubers; fruit; vegetable; fish; poultry or meat or eggs; legumes; milk and dairy products; confectionery and snacks; plain water and beverages; fast food; fat, oil, sugar, and salt. The FFQ were pre-tested among adolescents within the age range. During the interview sessions, respondents were asked to recall the frequency and quantity intake of the selected food items in the past three months. Household measurement tools for example teaspoon, tablespoon, cup, glass, and bowl as well as picture album were displayed by the interviewers during interview sessions to reduce recall bias.

Then the food items were re-categorized into new food groups according to the specific recommendations from Malaysian Dietary Guidelines 2010 [11] and the characteristics or functions of the foods. Therefore, 12 food groups namely refined cereals and grains; whole grains, cereals, and tubers; poultry, meat, eggs and seafood; fish; legumes; fruits; vegetables; milk and dairy products; sugar added beverages; confectionery and snacks; fast food; and food high in fat, oil, sugar, salt were re-organized from the original FFQ. Cereals and grains food group was divided into “refined cereals and grains” and “whole grain cereals and grains” because of the difference of food characteristics. 

**Figure 1:**
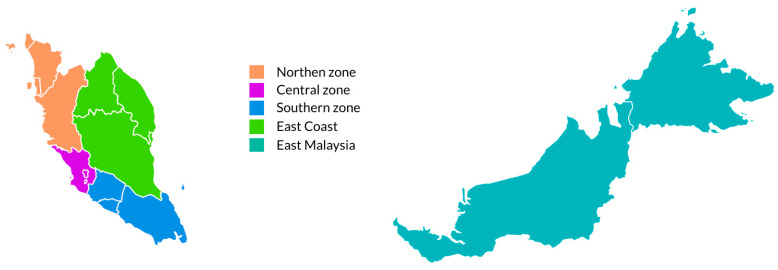
Locality of schools based on geographical areas.

In this study, the locality of schools was determined according to the geographical areas (Northern zone, Central zone, Southern zone, East Coast and East Malaysia). All of the states and federal territories were grouped into zones as shown in Figure 1. The northern zone consists of Perlis, Kedah, Pulau Pinang, and Perak. The states in Centre zone are Selangor, Putrajaya Federal Territory, and Kuala Lumpur Federal Territory. Southern zone covers Malacca, Negeri Sembilan, and Johor. East Coast comprises of the states of Pahang, Terengganu, and Kelantan. Sabah, Labuan Federal Territory and Sarawak are the states in East Malaysia. Types of school were categorized into Primary and Secondary school. Ethnicities of the respondents were grouped into three categories which were Malay, Chinese, or Indian, Indigenous from East Malaysia or others. 

The frequency of eating snacks and eating out from home in a week was coded as "four and more times”, “one to three times,” and “Never” based on the responses to the two multiple-choice questions: “How often do you have snacks in a week?” and “How often do you eat out in a week? Not including eating in school and hostel.” Self-perceived weight status of adolescents was assessed by a multiple-choice question: “At the present time, you think you are…” and followed by five answers of “significantly underweight,” “underweight,” “has appropriate body weight,” “overweight,” and “obese.” Then the responses were recorded as “underweight,” “appropriate body weight,” and “overweight or obese.” Food label reading habit was assessed by a multi-choice question: “Do you read food label when buying or receiving food/drink?” There were three responses provided for this question: “yes, every time,” “yes, sometimes,” and “no”.

### 2.3. Statistical Analysis

Data analyses were performed using SPSS version 20 (SPSS IBM, NY, USA). Weighing was applied to take into consideration of the complexity of the study design and non-responsive rate. Descriptive statistics were used to describe the socio-demographic characteristics and eating habits among adolescents. Dietary patterns were identified based on the daily frequency intake of each of the 12 food groups using exploratory factor analysis. Kaiser-Meyer-Olkin test (KMO) measurement of sample adequacy greater than 0.6 and the Bartlett test of Sphericity (BTS) of *p* < 0.05 were used to assess data adequacy for factor analysis. Factors were rotated with orthogonal (varimax method) rotation for creating independent factors and to improve interpretability between the factors. The Scree plot was used to determine the number of factors with an Eigen value of >1.0. Dietary pattern with Eigen value ≤ 1.0 were removed. Food groups with factor-loading greater than 0.30 were retained as the identified dietary patterns. 

The identified dietary patterns were labeled according to the characteristics of the food groups. Meanwhile, a summary score for each identified dietary pattern was derived. A high factor score for a dietary pattern indicates a high intake of the foods from the dietary pattern. The factor scores were then used in complex sample general linear model (GLM) analysis to examine the associations between various dietary patterns and the independent variables. All statistical analyses were carried out at 95% confidence interval or *p*-value < 0.05. 

## 3. Results

### 3.1. Socio-Demographic Characteristics and Dietary Habits of Respondents

In total, 2013 respondents completed the Food Frequency Questionnaire (FFQ) with a response rate of 96.4%. Table 1 shows the socio-demographic characteristics and dietary habits of respondents. In general, we have taken into account one-fourth from Central zone, 51.7% boys, 60.9% secondary school students, and 60.8% Malays. Only 5% of them did not eat snacks but most of them ate one to three times of snacks in a week. On the other hand, 11.9% of them did not eat out but three out of four of them were eating out from home one to three times in a week. In general, only one-third of them ate breakfast daily and 44.6% of them ate lunch daily in a week. Besides that, 39.5% of the respondents perceived that they had appropriate body weight while 32.7% of them thought that they were underweight, and 27.8% of them had an overweight or obese self-perception. Majority of the respondents practiced food label reading habit, 33.0% of them read every time when they made purchase and 49.7% of them read the label sometimes when they made purchase.

### 3.2. Food Group and Food Items in FFQ

Table 2 shows that 136 food items in FFQ which were categorized into 12 food groups. The frequency intake of 12 food groups was used to define dietary patterns. 

### 3.3. Dietary Patterns of the Respondents

Three dietary patterns were identified by analyzing 12 food groups and categorized them into unhealthy dietary pattern, healthy dietary pattern, and alternative proteins dietary patterns with Kaiser-Meyer-Olkin test (KMO) of 0.883, Bartlett test of Sphericity (BTS) of *p* < 0.001 (Table 2). Unhealthy dietary pattern was derived from foods with high sugar content, oil or fat, salt, and processed food. Healthy dietary pattern consisted of foods rich in nutrients, fibers, and protein. Alternative proteins dietary pattern was mainly foods from milk and dairy products as well as legumes and beans. The mean scores for the unhealthy dietary pattern is −0.040, healthy dietary pattern is 0.043, and alternative proteins dietary pattern is 0.057 (Table 3). 

Table 4 shows that none of the food group was excluded from the dietary pattern (factor-loading > 0.30). The unhealthy dietary pattern showed a combination of local foods and western foods with the characteristics of high in fat, sugar, and salt. The healthy dietary pattern mainly consisted of healthy foods such as vegetables, fruits, fish, and whole grains, cereals, and tubers. Milk and dairy products, legumes and soy-based products, were labeled as alternative proteins dietary pattern. These three dietary patterns explained that 54.2% of the total variations are 23.6% for the unhealthy dietary pattern, 15.3% for the healthy dietary pattern, and 15.6% for alternative proteins dietary pattern.

### 3.4. Associated Factors of Dietary Pattern Scores Among Respondents

The general linear models show that unhealthy dietary pattern was significantly associated with the locality of schools, sex, ethnicity of the adolescents, frequency of snacks intake, frequency of eating out, breakfast intake, self-perceived weight status, and food label reading habit. Alternative proteins dietary pattern was significantly associated with school category, ethnicity of the respondents, and breakfast intake. Meanwhile, a healthy dietary pattern was significantly associated with the locality of schools, ethnicity, school category, and frequency of eating out (Table 5).

## 4. Discussion

There were three dietary patterns (unhealthy, healthy, and alternative proteins) identified in this study. These food patterns were comparable to the food patterns analyzed by other studies among similar target groups. The unhealthy dietary pattern identified in this study were similar with the dietary pattern labeled as “Western pattern” from Iran [10], Brazil [17], and Korea [18]. The healthy dietary pattern from our findings was comparable with their “healthy dietary pattern” or “mixed dietary pattern” in previous studies carried out in Iran and Brazil [10,17]. Although the alternative proteins dietary pattern identified in this study was different from the findings from other countries, it was consistent with a local study in the Selangor state [15]. This finding may be due to the food items from alternative proteins dietary pattern are commonly sold in the school environment, for example school canteens, stores, and vendor machines.

Overall, there was a significant association between ethnicity and the three identified dietary patterns. Malay adolescents showed the highest factor scores for the unhealthy dietary pattern. Whereas Chinese or Indian adolescents showed the highest factor scores for healthy and alternative proteins dietary patterns compared to Malay and Indigenous people from East Malaysia. These findings were consistent with a previous local study in the state of Kelantan that revealed that Chinese adolescents exhibit a healthier food pattern than Malay adolescents [16] and another previous local study found that Malay adolescents had significant higher prevalence of poor diet quality than Indian adolescents [19]. The ethnic difference in dietary patterns may be due to difference in the social and cultural context of the ethnicities related to food choices [20]. 

Besides socio-cultural factors, food purchasing preferences were different across the ethnicities. Malays are primarily Muslims and require Halal certification for the food purchased [21]. We assume that Malay adolescents tend to have a less diverse diet compared to other ethnicities of adolescents because of this requirement. In addition, a review study revealed that adolescent’s low socio-economic status may be associated with poorer dietary patterns compared to higher adolescents with higher socio-economic status [22]. Therefore, this study suggests further research to investigate in depth about the modifiable factors of food choices such as belief of health benefits of healthy foods and affordability of purchasing healthy foods in Malay adolescents to improve the quality of diet. 

The present findings show significant associations between the locality of schools and unhealthy dietary patterns as well as alternative proteins dietary patterns. Adolescents from East Malaysia showed the highest factor score of unhealthy dietary pattern whereas adolescents from the Centre zone showed the highest factor scores of alternative proteins dietary pattern. The unhealthy dietary pattern in East Malaysia may be caused by two different possible factors. First, the development of fast food restaurants in East Malaysia, especially in the urban areas may have changed the traditional dietary pattern of adolescents to unhealthy dietary pattern. According to the findings from Ibrahim, the development of fast food restaurants in Sarawak were growing rapidly after inception of the Franchise Development Program in 1992 [23]. Second, many of the adolescents with lower socio-economic status in the rural areas in East Malaysia consumed unhealthy foods such as fried noodles, fried banana, doughnuts, chocolate drinks as breakfast because they were more affordable for them [24]. 

Meanwhile, adolescents from the Central zone (i.e., Kuala Lumpur, the nation’s capital and its surrounding areas—which have the highest levels of urbanization in the country) were more likely to adopt alternative protein sources, which mostly consist of dairy and plant-based protein products. This finding is supported by a previous study in South East Asian countries, which revealed that children residing in urban areas tend to consume more dairy products compared to children in rural areas [25]. It is possible that the marketing efforts of food and beverage companies are intensified in the Central zone, where large food retail stores (hypermarkets, supermarkets or departmental stores and shops) are readily accessible. These larger chains tend to stock a wider range of healthier foods—that also often cost more. Urban families in general are of a higher socioeconomic status compared to those residing in rural areas [26], which means more urban parents are aware of the need to (and can afford to) purchase these healthy foods for their children. It would thus be beneficial to coordinate and boost the supply chain between local dairy or plant-based protein processors and retailers in rural areas in order to provide more affordable products to the households in rural areas.

The findings from our study indicate that adolescents from primary schools showed the highest factors scores of healthy dietary pattern and alternative proteins dietary pattern. Our findings were supported by an Australian study that revealed that the quality of dietary intake habits tends to decrease with increasing age [27]. According to previous studies, parents guided adolescents to make healthy food choices decision whereas peers always shared unhealthy food items such as sugar-sweetened beverages and junk food particularly during school recess [28,29] and studies also found that healthy dietary pattern was associated with children below 10 years [30]. We assumed that adolescents from primary school (aged between 10 to 12 years) in this study were also more likely to practice healthy dietary pattern compared to adolescents from secondary school (aged between 13 to 17 years). Therefore, we hypothesized that a higher influence from parents and a less negative influence from peers in dietary practices among adolescents from primary schools could be the cause of having a healthy diet [31]. 

There were several limitations to be addressed in this study. First, response bias may occur in this study because of misunderstanding of the dietary questions or the respondents avoid reporting their unhealthy dietary habits, for example frequency of eating snacks and foods out of home even this study had been carried out anonymously [32]. Second, a set of unvalidated FFQ was used in this study. However, this FFQ was developed by the expert panel and pre-tested among adolescents. Third, this study was limited by the use of factor analysis, which requires some subjective interpretation of the results. However, factor analysis was the most widely used method to identify dietary patterns [7,9,33]. Apart from these limitations, this study has several strengths. First, face-to-face interviews of FFQ were conducted by trained nutritionists. In order to collect more accurate information regarding to dietary intake pattern of adolescents, frequency of intake of 136 food items was captured through open-ended questions. In addition, the use of household measurement tools and picture album during interview sessions helped to enhance the accuracy of the food intake and avoid respondents’ under or over reporting. 

This study showed that dietary patterns are associated with ethnicity, locality of schools, and types of school in adolescents. Adolescents who are Malay, living in East Malaysia, and attending secondary school tend to practice unhealthy dietary pattern. Public health policy-makers and programme managers should take these findings into consideration during programme planning and implementation of intervention for improving quality of diet and general health status in adolescents. We recommend ethnicity-specific and geographical-area-specific strategies to promote healthy eating habits among Malay adolescents and balance diet among Indigenous people in the East Malaysia. 

## 5. Conclusions

Three major dietary patterns were identified among the adolescents in Malaysia. This study found that Malay adolescents, living in East Malaysia, and attending secondary school adapted to unhealthy dietary practice. Therefore, ethnicity-specific and geographical-area-specific strategies are suggested to improve dietary patterns of adolescents in Malaysia. 

## Figures and Tables

**Table 1:** Socio-demographic characteristics and dietary habits of the respondents.

Characteristics	Count (n)	% (95% CI)
Locality of schools		
Northern	500	19.1 (17.6–20.8)
Centre	354	26.7 (23.2–30.5)
Southern	389	19.3 (17.7–21.1)
East coast	342	11.9 (10.7–13.1)
East Malaysia	428	23.0 (20.9–25.2)
Types of school		
Primary	646	39.1 (30.9–47.9)
Secondary	1367	60.9 (52.1–69.1)
Sex		
Boys	1006	51.7 (48.4–55.0)
Girls	1007	48.3 (45.0–51.6)
Ethnicity		
Malay	1309	60.8 (55.5–65.9)
Chinese/Indian	423	23.5 (18.7–29.1)
Indigenous people from East	281	15.7 (13.0–18.8)
Malaysia/others		
Snacks intake per week		
≥ 4 times	526	26.1 (23.7–28.7)
1-3 times	1376	68.9 (66.0–71.6)
Never	97	5.0 (3.8–6.5)
Eating out per week		
≥ 4 times	313	15.9 (13.7–18.3)
1-3 times	1446	72.3 (70.0–74.4)
Never	244	11.9 (10.0–14.1)
Breakfast intake per week		
Every day	580	30.1 (27.2–33.1)
1-6 days	1248	60.7 (57.3–64.0)
Never	179	9.2 (7.7–11.1)
Lunch intake per week		
Every day	892	44.6 (40.0–48.4)
1-6 days	1060	53.0 (49.2–56.7)
Never	51	2.4 (1.8–3.3)
Self-perceived weight status		
Underweight	623	32.7 (29.7–35.8)
Overweight or obese	554	27.8 (24.8–31.0)
Appropriate	830	39.5 (36.8–42.4)
Food label reading habit		
Yes, every time	445	33.0 (30.0–36.0)
Yes, sometimes	690	49.7 (46.3–53.1)
No	224	17.3 (14.9–20.0)

**Table 2:** Food groups and food items in Food Frequency Questionnaire (FFQ).

Food Groups	Food Items
Cereals, grains, cereals products, and tubers	White rice, white bread, fried rice, *nasi lemak*, instant noodle, chicken rice, rice vermicelli, *roti canai*, tubers, rice noodle, buns, wheat noodle, sweet corn, breakfast cereals, chocolate-flavored biscuits, cream cracker, porridge, pasta, *murtabak*, Marie biscuit, wholegrain bread, *nasi kerabu*, chapati, *nasi dagang*, thosai
Poultry or meat or eggs	Chicken, chicken eggs, sausage, anchovies, shrimp, fish/shrimp/squid/crab/chicken balls, squid, beef, crab, salted eggs, cockle flesh, mutton, pork, Dim sum, quail eggs, duck meat, duck eggs
Legumes	Soya milk, fried groundnut, dhal, melon seeds, tofu, tofu pudding, *tempe*, *kacang puteh*, broad beans
Fish	Whole marine fish, sliced marine fish, canned fish, whole freshwater fish, sliced freshwater fish
Milk and dairy products	Cultured drinks, UHT milk, fresh milk, cheese, milk powder
Fruit and vegetable	Apple, banana, orange, watermelon, mango, grapes, dried fruits, papaya, guava, *lai*, pineapple, honeydew, local sweet orange, rambutan, lychee, durian, *mata kucing*, starfruit, mangosteen
Vegetable	Green leafy vegetables, flowered/flower buds vegetables, carrot, podded vegetables, cucumber, tomato
Plain water and beverages	Plain water, malted drinks, ready to drink tea, carbonated drinks, various flavor cordial drinks, fruit juice, pre-mixed drink, ice blend, ready to drink coffee
Confectionery and snacks	Candy, curry puff, fried banana, dairy ice cream, fried fish crackers, crispy crackers, chocolate bar, doughnut, cake, potato chips, cream cookies, ice beans/*cendol*, *pau, cekodok*, non-dairy ice cream, tuber/banana crisps, *kuih lapis*, fish/shrimp crackers, fried spring rolls, prawn fritter, *char kuey*, *kuih keria*, Chinese doughnut, *kuih vadai*
Fast food	Fried chicken, burger, French fries, nugget, pizza, mashed potato, coleslaw
Fat, oil, sugar, and salt	Sugar, soy sauce, chili sauce, coconut jam, mayonnaise, tomato sauce, margarine, butter, peanut butter, fruit jam

**Table 3:** Dietary patterns of adolescents in Malaysia.

Dietary Pattern	Mean Factor Scores	Lower	Upper	Total Variation Explained (%)
Unhealthy	−0.040	−0.126	0.045	23.6
Healthy	0.043	−0.041	0.128	15.3
Alternative proteins	0.057	−0.060	0.173	15.2

Kaiser-Meyer-Olkin test (KMO) = 0.883; Bartlett test of Sphericity (BTS) of *p* < 0.001; Total variation explained equal to 54.0% (23.6% from unhealthy pattern, 15.3% from healthy pattern, and 15.2% from alternative proteins pattern).

**Table 4:** List of factor-loading of dietary patterns.

Food Groups	Dietary Patterns		
	Unhealthy	Healthy	Alternative Proteins
Sugar added beverages	**0.722**	−0.089	0.191
Fat, oil, sugar and salt	**0.712**	0.064	−0.059
Confectionery and snacks	**0.673**	0.134	0.420
Refined grains and cereals	**0.588**	0.353	0.255
Poultry, meat, eggs, and seafood	**0.606**	0.334	0.128
Fast food	**0.558**	−0.025	0.517
Vegetables	0.064	**0.719**	0.164
Fish	0.479	**0.578**	−0.285
Fruits	0.200	**0.553**	0.476
Whole grains, cereals, and tubers	−0.019	**0.544**	0.219
Milk and dairy products	0.069	0.182	**0.754**
Legumes and soy-based products	0.204	0.289	**0.550**

Bold font: factor-loading > 0.30.

**Table 5:** Factors associated with dietary pattern among respondents.

Factors	Factor Scores	95% CI		F Value	*p* Value
		Lower	Upper		
Unhealthy dietary pattern ^1^					
Locality of schools				9.774	<0.001
Northern	−0.379	−0.570	−0.189		
Centre	−0.480	−0.716	−0.244		
Southern	−0.561	−0.746	−0.377		
East coast	0.070	−0.205	0.344		
East Malaysia	0.119	−0.158	0.396		
Sex				4.540	0.034
Male	−0.174	−0.316	−0.032		
Female	−0.319	−0.483	−0.154		
Ethnicity				44.517	<0.001
Malay	0.079	−0.048	0.206		
Chinese/Indian	−0.588	−0.738	−0.439		
Bumiputra/others	−0.230	−0.544	0.084		
Snacks intake per week				14.717	<0.001
≥4 times	−0.028	−0.206	0.149		
1–3 times	−0.108	−0.245	0.030		
Never	−0.603	−0.813	−0.393		
Eating out per week				3.747	0.025
≥4 times	−0.049	−0.293	0.195		
1–3 times	−0.275	−0.400	−0.149		
Never	−0.416	−0.589	−0.243		
Self-perceived weight status				3.556	0.030
Underweight	−0.127	−0.306	0.052		
Overweight/obese	−0.381	−0.569	−0.193		
Appropriate	−0.231	−0.375	−0.087		
Food label reading habit				4.375	0.014
Yes, every time	−0.167	−0.324	−0.010		
Yes, sometimes	−0.348	−0.492	−0.204		
No	−0.224	−0.455	0.006		
Healthy dietary pattern ^2^					
Types of school				16.177	<0.001
Primary school	0.269	0.132	0.407		
Secondary school	−0.057	−0.163	0.050		
Ethnicity				3.106	0.046
Malay	0.005	−0.096	0.106		
Chinese/Indian	0.212	0.081	0.342		
Bumiputra/others	0.102	−0.106	0.309		
Alternative proteins dietary pattern ^3^					
Locality of schools					
Northern	−0.133	−0.269	0.003	2.737	0.029
Centre	0.328	0.081	0.574		
Southern	−0.111	−0.265	0.042		
East coast	−0.136	−0.377	0.105		
East Malaysia	0.045	−0.098	0.188		
Ethnicity				4.715	0.010
Malay	0.052	−0.075	0.180		
Chinese/Indian	0.149	0.043	0.255		
Bumiputra/others	−0.207	−0.437	0.023		
Types of school				11.828	0.001
Primary school	0.155	0.015	0.294		
Secondary school	−0.158	−0.273	−0.043		

^1^ Types of school, breakfast intake per week, and lunch intake per week were removed from the univariate GLM model for unhealthy pattern. ^2^ Locality of schools, sex, snacks intake per week, eating out per week, breakfast intake per week, lunch intake per week, self-perceived weight status and food label reading habit were removed from the univariate GLM model for healthy pattern. ^3^ Sex, snacks intake per week, eating out per week, breakfast intake per week, lunch intake per week, self-perceived weight status and food label reading habit were removed from the univariate GLM model for alternative proteins pattern.

## Abbreviations

ANS: Adolescent Nutrition Survey; FFQ: Food Frequency Questionnaire; KMO: Kaiser-Meyer-Olkin test; BTS: Bartlett test of Sphericity; GLM: general linear model.
